# Uncovering Bifidobacteria via Targeted Sequencing of the Mammalian Gut Microbiota

**DOI:** 10.3390/microorganisms7110535

**Published:** 2019-11-06

**Authors:** Gabriele Andrea Lugli, Sabrina Duranti, Christian Milani, Leonardo Mancabelli, Francesca Turroni, Douwe van Sinderen, Marco Ventura

**Affiliations:** 1 Laboratory of Probiogenomics, Department of Chemistry, Life Sciences, and Environmental Sustainability, University of Parma, 43124 Parma, Italysabrina.duranti@unipr.it (S.D.); christian.milani@unipr.it (C.M.); leonardo.mancabelli@genprobio.com (L.M.); francesca.turroni@unipr.it (F.T.); 2 Microbiome Research Hub, University of Parma, 43124 Parma, Italy; 3 APC Microbiome Institute and School of Microbiology, Bioscience Institute, National University of Ireland, T12 YT20 Cork, Ireland; d.vansinderen@ucc.ie

**Keywords:** genomics, metagenomics, microbiota, *Bifidobacterium*

## Abstract

Bifidobacteria are among the most prevalent gut commensals in mammals, playing crucial functional roles that start from their early colonization of the infant gastrointestinal tract and last throughout the life span of their host. Metagenomic approaches have been employed to unveil the genetic features of bifidobacteria in order to understand how they participate in the correct development of a healthy microbiome. Nevertheless, their low relative abundance in many environmental samples may represent a major limitation for metagenomics approaches. To overcome this restriction, we applied an enrichment method that allows amplification of bifidobacterial DNA obtained from human or animal fecal samples for up to 26,500-fold, resulting in the metagenomic reconstruction of genomes belonging to bifidobacterial strains, present at very low abundance in collected samples. Functional predictions of the genes from these reconstructed genomes allows us to identify unique signatures among members of the same bifidobacterial species, highlighting genes correlated with the uptake of nutrients and adhesion to the intestinal mucosa.

## 1. Introduction

The human body, as well as that of non-human animals, is inhabited by a plethora of microbial species that reside in specific microbial communities in and on their host [1]. The gastrointestinal microbial community, also known as gut microbiota, represents the most numerous host-associated community, with an estimated 10^14^ bacterial cells located in the large intestine [2]. Here, the microbial population exerts many important activities to sustain host health, including a breakdown of otherwise indigestible food components, pathogen protection, promotion of host cell differentiation, and stimulation/modulation of the host immune system [3,4]. For this reason, the gut microbiota is the most scrutinized host-associated community in terms of large scale metagenomic studies [5,6].

Bifidobacteria are among the first microbes colonizing the human gastrointestinal tract (GIT) as well as that of other mammals [6,7]. The presence of members of this genus is positively associated with the health status of the host [8]. In this regard, several species of the genus *Bifidobacterium*, i.e., *Bifidobacterium adolescentis*, *Bifidobacterium animalis*, *Bifidobacterium bifidum*, *Bifidobacterium breve* and *Bifidobacterium longum*, are known for their health-promoting or probiotic properties [9,10,11]. As mentioned above, bifidobacteria represent one of the dominant members of the core infant gut microbiota, while, from infancy to adulthood, fluctuations of the gut microbiota composition generally result in lower bifidobacterial levels (2–14% relative abundance) [12]. Thus, reconstruction of a complete bifidobacterial genome from the gut microbiota of adults can be quite a challenge due to the relatively low abundance of members of this genus.

In recent years, next-generation sequencing technologies have allowed in depth exploration of the gut microbiota composition, which has generated a wealth of genomic data [13]. The whole metagenome sequencing (WMS) methodology provides genetic information about those microorganisms present in a complex microbial consortium such as the gut microbiota [14,15]. Nonetheless, the ability to obtain a properly reconstructed genome sequence of a given bacterial strain is strictly correlated with the relative abundance of that particular microorganism within the sample as well as with the sequencing depth of the applied next-generation sequencing technology. To obtain a more complete view of the microbial diversity in a given sample, additional tools have been developed, such as targeted genome sequencing, allowing genome sequence enrichment of specific microbial species via hybridization to environmental DNA [16,17,18]. The reconstruction of the genetic material obtained through WMS, enhanced by targeted sequencing, provides access to the genome content of specific microorganisms without the need to isolate such bacteria, for example beneficial microbes that reside in the gut of mammals at very low relative abundance [19].

The focus of the current study was to apply targeted WMS in order to amplify the DNA of gut commensal species belonging to the genus *Bifidobacterium* among the gut microbiota of mammals. For this purpose, we sequenced and analyzed fecal samples collected from a child and a pygmy hippopotamus (*Choeropsis liberiensis*) possessing a varying abundance of bifidobacterial species. Thus, targeted sequencing of bifidobacterial DNA was performed allowing the reconstruction of bifidobacterial genome sequences in both samples.

## 2. Materials and Methods

### 2.1. DNA Extraction

DNA was extracted using the QIAmp DNA Stool Mini Kit following the manufacturer’s instructions (Qiagen, Hilden, Germany) from fecal samples collected from previous studies [20,21]. DNA concentration and purity were then determined employing a Picodrop microtiter Spectrophotometer (Picodrop, Hinxton, UK).

### 2.2. Identification of Bifidobacteria by 16S rRNA Gene Sequencing

Partial 16S rRNA gene sequences were amplified from extracted DNA using primer pair Probio_Uni/Probio_Rev, targeting the V3 region of the 16S rRNA gene sequence [22]. Sequencing was performed on a V3 600 cycle flow cell using a MiSeq (Illumina, San Diego, CA, USA) at the DNA sequencing facility of GenProbio srl (Available online: www.genprobio.com). DNA amplification was performed employing the Probio_Uni/Probio_Rev primer pair followed by purification with magnetic AMPure XP beads. Barcode annealing was performed employing Illumina Nextera XT Index Kit v2, followed by purification with magnetic AMPure XP beads and quantification with Qubit assay. Samples were then loaded with 20% of Phix. Following sequencing, fastq files were processed using a custom script based on the QIIME software suite [23]. Paired-end read pairs were assembled to reconstruct the complete Probio_Uni/Probio_Rev amplicons. Quality control retained sequences with a length between 140 and 400 bp and a mean sequence quality score of >20, while sequences with homopolymers of >7 bp and mismatched primers were omitted. In order to calculate downstream diversity measures (alpha and beta diversity indices, Unifrac analysis), 16S rRNA Operational Taxonomic Units (OTUs) were defined at 100% sequence homology using DADA2 [24]. OTUs not encompassing at least 2 sequences of the same sample were removed. All reads were classified to the lowest possible taxonomic rank using QIIME2 [25] and a reference dataset from the SILVA database release 132 [26].

### 2.3. Bifidobacterial ITS Sequencing

The Internal Transcribe Spacer (ITS) sequences of bifidobacteria were amplified from extracted DNA using primer pair ProbioBif-ITS_Fw and ProbioBif-ITS_Rev, which targets the variable region between the 16S rRNA and 23S rRNA gene sequences [27]. Sequencing was performed on a V3 600 cycles flow cell using a MiSeq (Illumina) at the DNA sequencing facility of GenProbio srl (Available online: www.genprobio.com) according to a previously described protocol [27]. Samples were then loaded with 20% of Phix. Following sequencing, fastq files were processed using a custom script based on the QIIME software suite [23]. Quality control retained sequences with a length between 100 and 400 bp and mean sequence quality score of >20, while sequences with homopolymers of >7 bp in length and mismatched primers were removed. In order to calculate downstream diversity measures (alpha and beta diversity indices, Unifrac analysis), ITS Operational Taxonomic Units (OTUs) were defined at 100% sequence homology using uclust [28]. All reads were classified to the lowest possible taxonomic rank using QIIME2 [25,27] and a reference dataset, consisting of an updated version of the bifidobacterial ITS database [20,27].

### 2.4. Experimental Design of myBaits for The Targeted WMS

A selection of 62 bifidobacterial type strains were used to build the baits (Table S2). Genome sequences of selected strains were sent to Arbor Biosciences (Ann Arbor, MI, USA) where the myBaits^®^ custom kit was designed. Thus, biotinylated RNA baits representative of the entire 62 nuclear genomes were produced. Resulting bifidobacterial baits were used in the targeted WMS of the analyzed samples of gut of mammals.

### 2.5. Bifidobacterial DNA Targeted Enrichment

In-solution sequence capture of bifidobacterial DNA was carried out using the MyBaits® custom kit (Arbor Biosciences) according to the manufacturer’s protocol (MyBaits® user manual 4.01, 2018). Enriched libraries were sequenced using the TruSeq Nano DNA LT sample preparation kit (Illumina) according to the manufacturer’s instructions. Two hundred ng of DNA was used as an input from each sample for library preparation. The isolated DNA underwent mechanical fragmentation by means of a Bioruptor, adapter ligation and amplification. The ready-to-go libraries were pooled equimolarly, denaturated and diluted to a sequencing concentration of 1.5 pM. Sequencing was performed on NextSeq 550 instrument (Illumina), according to the manufacturer’s instructions, using the 2 × 150 bp High Output sequencing kit, and spike-in of 1% PhiX control library.

### 2.6. Metagenomic Analyses

Data set quality of each sample was improved by means of a filtering step to obtain only high-quality reads (minimum mean quality score 20, window size 5, quality threshold 25 and minimum length 100) using the fastq-mcf script (Available online: https://github.com/ExpressionAnalysis/ea-utils/blob/wiki/FastqMcf.md). Collected filtered reads were subjected to de novo metagenomic assemblies using SPAdes v3.12 [29] with default parameters of the metagenomic flag option (-meta) coupled with minimum k-mer sizes of 21, 33, 55 and 77. Resulting contigs were taxonomically classified based on homology searches defined by means of RAPSearch2 (Reduced Alphabet based Protein similarity Search 2) [30] employing the RefSeq NCBI databases. The above-mentioned steps, starting from the paired-end read filtering to the taxonomic classification of the assembled contigs were independently performed by employing the METAnnotatorX pipeline [31].

### 2.7. Comparative Genomics

Open reading frames (ORFs) of both reconstructed genomes were predicted with Prodigal [32] and annotated by means of MEGAnnotator software [33]. Two pan-genome calculations were performed using the pan-genome analysis pipeline PGAP [34], including ORFs of 51 *B. adolescentis* and 280 *B. longum* genomes collected from the NCBI database and genomes of the two reconstructed strains AS-ADO and IS-LON (Table S3). Each predicted proteome of a given bifidobacterial strain was screened for orthologues against the proteome of every collected genome by means of BLAST analysis [35] (cutoff: E value of 1 × 10^−5^ and 50% identity across at least 80% of both protein sequences). The resulting output was then clustered into protein families by means of MCL (graph theory-based Markov clustering algorithm) [36], using the gene family (GF) method. Average Nucleotide Identity (ANI) values were calculated using the program JSpecies version 1.2.1 [37] between reconstructed genome sequences and type strains of *B. adolescents* and *B. longum* subsp. *longum* species.

### 2.8. Data Availability

Raw sequences of 16S rRNA gene and bifidobacterial ITS profiling experiments are accessible through SRA study BioProject PRJNA574035, while shotgun metagenomics data are accessible through SRA study BioProject PRJNA574033.

## 3. Results and Discussion

### 3.1. Identification of Bifidobacteria in the Gut of Mammals

**Figure 1:**
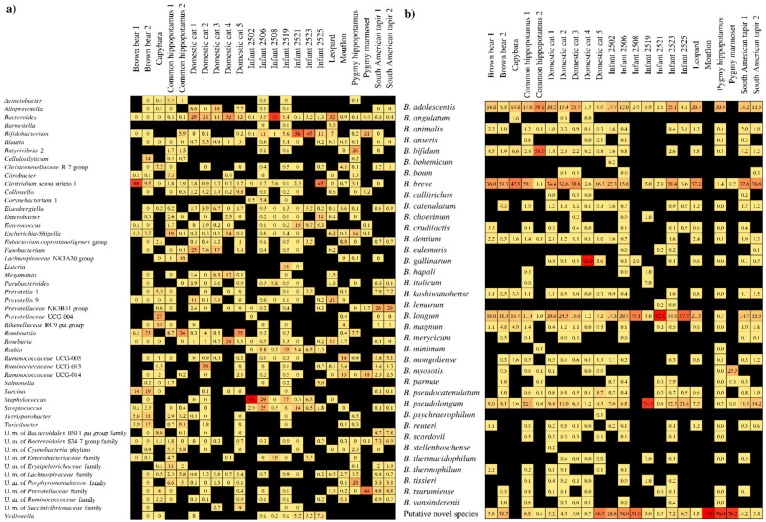
Bifidobacterial profiling of 23 fecal samples of human and animals. Panel (**a**) displays the relative abundance of each genus by means of 16S rRNA microbial profiling. Only genera that display at least one sample with a relative abundance of at least 5% were included in the heat map. Panel (**b**) shows the relative abundance of each species belonging to the genus *Bifidobacterium* by means of ITS bifidobacterial profiling. Only species that display at least 0.01% of the total amount of the sequencing data were included in the heat map.

In order to investigate the bifidobacterial composition of the mammalian gut microbiota, we inspected the taxonomic composition of the bacterial community harbored by seven human beings and 16 non-human animals through 16S rRNA-based profiling (Table S1). Illumina-mediated 16S rRNA microbial profiling produced more than one million sequence reads with an average of 16,495 filtered reads per sample (Table S1). Nucleotide sequences were grouped in clusters of operational taxonomic units (OTUs) and then taxonomically classified. At the genus level, this analysis showed that the bifidobacterial abundance ranged up to 55.5%, yet with eight samples in which bifidobacteria were undetectable (Figure 1).

In order to characterize the bifidobacterial population at the species level, in conjunction with 16S rRNA-based profiling, we employed bifidobacterial Internally Transcribed Spacer (ITS) profiling analysis [28], resulting in an average of 4488 high-quality filtered reads per sample (Table S1). The latter approach allowed us to cluster sequences in OTUs followed by their taxonomic classification at a (sub)species level [28]. Collected data were employed to evaluate the distribution of bifidobacterial species across 23 samples, defined as prevalence (Figure 1). 

Bifidobacterial profiling allowed us to select the sample with the highest relative abundance of bifidobacteria, represented by an infant sample (IS) (55.5%) named “Infant 2521” (Figure 1), and one with the lowest bifidobacterial abundance, represented by the animal sample (AS) (0.2%) named “Pygmy hippopotamus” (Figure 1). Based on the ITS-mediated bifidobacterial taxonomic classification, the bifidobacterial population present in sample IS was composed of *B. longum* (91.3%), while that of AS of *B. adolescentis* (20.3%) and a putative novel species of the genus *Bifidobacterium* (75.5%). Accordingly, fecal samples of IS and AS were subjected to WMS to reconstruct the genome content of the bifidobacterial species highlighted.

### 3.2. Targeted Sequencing of the Bifidobacterium genus

One of the major limitations of the WMS approach is the inability to retrieve genomic information of bacterial species that are present at a very low relative abundance, resulting in genomes with a sequencing coverage lower than five, which is insufficient to allow a meaningful genome assembly. To overcome this issue, various strategies employ the capture and enrichment of targeted bacterial DNA prior to next generation sequencing [17,18]. In the field of microbiology, this approach has been successfully used for the enrichment of pathogens and viruses [16,17,38,39], usually in order to obtain DNA amplification of a single bacterial/virus strain. Instead, we designed genus-specific probes based on genomic data available for 62 publicly available type strains of the genus *Bifidobacterium*, in order to enrich fecal-derived DNA samples for genetic material corresponding to known bifidobacteria (see Material and Methods Section 2.4) (Table S2). Selected strains were chosen in order to avail of genomic sequence variability across the genus *Bifidobacterium* that would target only a limited number of bifidobacterial genomes.

WMS of such targeted bifidobacterial DNA from stool samples IS and AS produced approximately 45 million of paired-end reads with an average length of ~150 bp, which were analyzed through the METAnnotatorX pipeline [32]. A preliminary screening, based on the sequence reads, revealed marked increases in the relative abundance of bifidobacteria when compared to the 16S rRNA gene-based data of the original samples, i.e., 53.2% in AS and 70% in IS. Based on these data, IS, which was already rich in bifidobacteria, was enriched by 26%, while AS, which was very low in its bifidobacterial load, was enriched by more than 26,500-fold.

Based on these results, we observed that our strategy to enrich bifidobacterial genome sequences in WMS data was successful, working even better when the starting DNA sample was very low in bifidobacterial DNA content. Furthermore, this approach amplified the genus-specific DNA also in the sample already rich in bifidobacterial DNA in the original biological sample, resulting in a robust strategy to obtain even more targeted bacterial DNA from a complex matrix such as that of stool samples.

### 3.3. Bifidobacterial Genome Reconstruction

**Figure 2:**
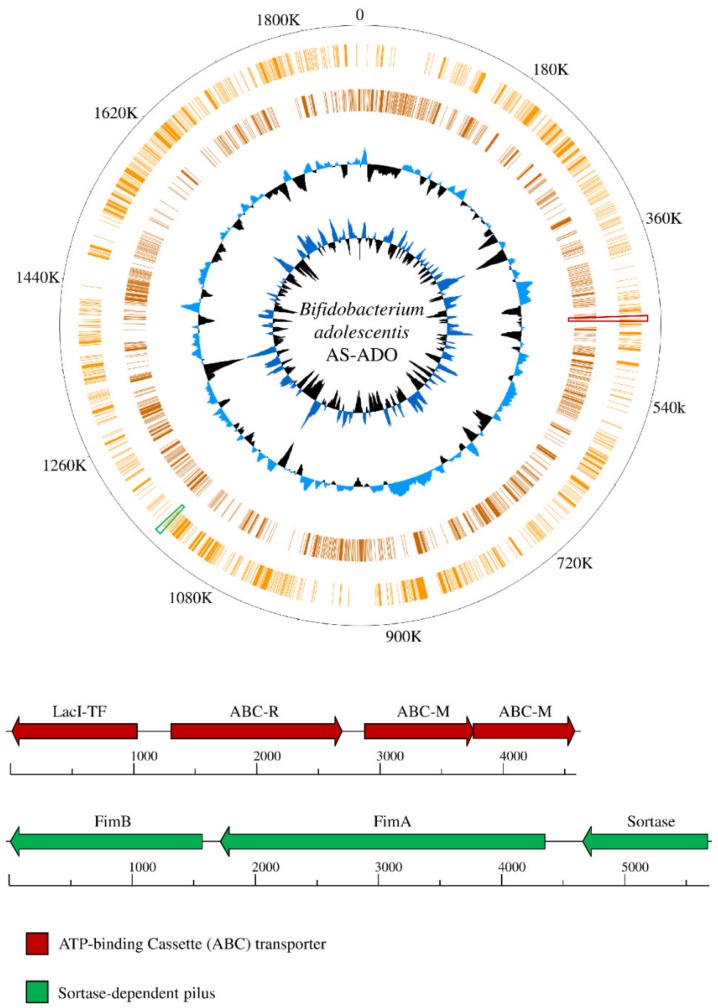
*Bifidobacterium adolescentis* AS-ADO unique loci. *B. adolescentis* AS-ADO reconstructed contig sequences are ordered based on the genome of the type strain *B. adolescentis* ATCC 15703 complete genome and its genes reported as a circular genome atlas (orange circles). Internal circles illustrate *B. adolescentis* AS-ADO GC% deviation and GC skew (G−C/G+C). Genetic maps exhibit two unique loci of *B. adolescentis* AS-ADO compared to *B. adolescentis* strains retrieved from the database. Loci positions in the genome are highlighted with the relative color. Each arrow indicates an open reading frames (ORF), whereas the length of the arrow is proportional to the length of the predicted ORF.

Metagenomic data was then assembled in silico, revealing 141 contigs with a length of > 5000 bp (43 in IS and 98 in AS) predicted to belong to the genus *Bifidobacterium* by means of taxonomical classification based on the proteome of each contig (Figure 2). Bifidobacterial contigs were subsequently classified at the species level unveiling a prevalence which was consistent with that obtained from ITS bifidobacterial profiling (Figure 1). In this context, all 43 IS-derived assembled bifidobacterial contigs were predicted to belong to the *B. longum* subsp. *longum* species, while the 89 AS-derived contigs were predicted to represent the *B. adolescentis* species. The two reconstructed bifidobacterial strains were named *B. adolescentis* AS-ADO and *B. longum* subsp. *longum* IS-LON and the size of their reconstructed genome was estimated to be 1.86 and 2.37 Mb, respectively (Table 1). Quality of the resulting genomes was guaranteed by the high coverage of the assembled bifidobacterial sequences, being in excess of one thousand for both genomes (Table 1).

To validate the taxonomic classification attributed to the reconstructed bifidobacterial contigs, average nucleotide identity (ANI) analysis was employed. Thus, the sequence of the two decoded genomes were compared with those of the type strains *B. adolescentis* ATCC 15703 and *B. longum* subsp. *longum* DSM 20088, highlighting that the reconstructed *B. adolescentis* AS-ADO and *B. longum* subsp. *longum* IS-LON displayed ANI values of 97.9% and 97.1%, respectively (Table 1). Notably, two bifidobacterial strains displaying an ANI value of >94% are considered to belong to the same species [40,41], validating the predicted taxonomic classification of the reconstructed bifidobacterial genomes.

Altogether, the taxonomic classification of the reconstructed bifidobacterial genomes highlights that the enriched bifidobacterial DNA, due to targeted enrichment prior to sequencing, corresponds to known bifidobacterial species predicted by ITS bifidobacterial profiling. Nevertheless, targeted amplification of sample AS was not able to capture DNA related to the putative novel species of the genus *Bifidobacterium*. Thus, this approach, based on the manufacturer’s protocol (see Materials and Methods Section 2.5), can be applied to any complex matrix in order to capture DNA that is strictly related to the bifidobacterial species used for the generation of the baits. Therefore, in order to enrich DNA from novel bifidobacterial strains, the targeted WMS approach has to be further improved by modifying the protocol for DNA capture.

### 3.4. Insights into The Genetics of The Reconstructed Bifidobacterial Strains

Gene prediction based on the reconstructed *B. adolescentis* AS-ADO and *B. longum* subsp. *longum* IS-LON genomes revealed 1549 and 2024 predicted protein-encoding open reading frames (ORFs), respectively (Table 1). Genes predicted from the reconstructed contigs were then employed, together with those belonging to publicly available genomes belonging to bifidobacterial strains of the same species, to perform comparative genome analyses involving 52 *B. adolescentis* and 281 *B. longum* strains (see Table S3 for the complete list of bifidobacterial strains retrieved from the NCBI genome database). A total of 8206 and 24,553 Cluster of Orthologous Groups (COGs) were identified from the analysis based on available *B. adolescentis* and *B. longum* genomes, respectively. Comparative genomic analyses allowed the identification of truly unique genes (TUGs) of both species, i.e., those genes that are present in one strain yet absent in any of the other examined. Discarding partial genes located at the edge of contigs and genes smaller than 300 nucleotides, *B. adolescentis* AS-ADO and *B. longum* subsp. *longum* IS-LON display 33 and 19 functional TUGs, respectively (Table 1).

Interestingly, detailed cataloguing of TUGs of *B. adolescentis* AS-ADO allowed the identification of genes, present only in AS-ADO, that may increase the ecological fitness of the strain based on their in silico predicted function. In this context, AS-ADO encodes a unique ATP-binding Cassette (ABC) transporter composed by three subunits, i.e., two permease proteins and a solute-binding protein predicted to be involved in maltose or trehalose uptake, flanked in the genome by a gene encoding a unique regulatory protein belonging to the *Lac*I family (Figure 2). This *B. adolescentis* specific gene cluster, which is homologous with that found in *Bifidobacterium moukalabense* DSM 27321, may enhance the ecological fitness of AS-ADO to grow in the gut of its host, the pygmy hippopotamus. Furthermore, the same strain harbors a gene encoding a unique large fimbrial protein and located between a gene specifying a small fimbrial protein and a sortase-encoding gene already classified in the *B. adolescentis* species [42]. This sortase-dependent pilus locus may improve the adhesion of cells to the intestinal mucosa of its host, thereby perhaps allowing or supporting persistence and colonization of AS-ADO.

In contrast, none of the unique genes of *B. longum* subsp. *longum* IS-LON was predicted to modify its ecological fitness based on current knowledge on homologous genes and protein domains in the available databases. Moreover, using 280 additional *B. longum* genomes available in public databases, we were able to confirm what had previously been observed through pan-genome analysis of 37 *B. longum* strains belonging to the human gut, highlighting a pan-genome that has reached a closed state [43]. Nevertheless, DNA enrichment of *B. adolescentis* AS-ADO allowed us to detect unique genetic signatures that were impossible to identify by means of a standard WMS strategy. Thus, targeted bifidobacterial WMS, when applied to samples in which the reconstruction of genomes would be difficult due to the low abundance of the specific genomic DNA in the starting material, and may allow insights into the unique features of novel bifidobacterial strains. 

## 4. Conclusions

Bifidobacteria are dominant gut commensals of mammals, playing a pioneering role during the initial bacterial colonization stage of the GIT during the first days after birth. Unveiling their genetic features is crucial to understand how they positively impact on the appropriate development of a healthy microbiome. Via targeted capture of bifidobacterial DNA from a human and an animal sample, we were able to enrich bifidobacterial DNA up to 26,500 times, resulting in the metagenomic reconstruction of genomes at low abundance in the collected samples. Predicted gene functions of such reconstructed genomes allowed the identification of unique genetic signatures among members of the same species, highlighting in one case the existence of genes for the presumed uptake of nutrients and the adhesion of cells to the mucosa of the large intestine.

The approach outlined in this work may be further improved by modifying the protocol for DNA capture, in order to enrich DNA from novel bifidobacterial strains or species. This procedure will assist in the exploration of (bifido)bacterial dark matter that is resident in the mammalian gut microbiota. Furthermore, adapting this procedure for DNA enrichment of other bacterial genera will allow us to shed light on such microbial dark matter.

## Figures and Tables

**Table 1:** General features of reconstructed bifidobacterial genomes.

Features	*B. adolescentis* AS-ADO	*B. longum* subsp. *longum* IS-LON
Biological origin	*Hippopotamus amphibius*	*Homo sapiens*
Average Coverage	1057	1855
Contigs	89	43
Genome length	1,857,949	2,366,427
Average GC percentage	59.44	59.85
Predicted ORFs	1549	2,024
ANI value (%, species)	97.9, *B. adolescentis* ATCC 15703	97.1, *B. longum* subsp. *longum* DSM 20088
TUGs	33	19

## Supplementary Materials

The following are available online at https://www.mdpi.com/2076-2607/7/11/535/s1, Supplementary Table S1. Quality-filtering table of 16S rRNA gene and ITS bifidobacterial profiling datasets. Supplementary Table S2. Bifidobacterial genomes used to design the myBaits. Supplementary Table S3. Bifidobacterial genomes used to perform comparative genomics analyses.
